# Succinate/NLRP3 Inflammasome Induces Synovial Fibroblast Activation: Therapeutical Effects of Clematichinenoside AR on Arthritis

**DOI:** 10.3389/fimmu.2016.00532

**Published:** 2016-12-07

**Authors:** Yi Li, Jia-Yi Zheng, Jian-Qun Liu, Jie Yang, Yang Liu, Chen Wang, Xiao-Nan Ma, Bao-Lin Liu, Gui-Zhong Xin, Li-Fang Liu

**Affiliations:** ^1^State Key Laboratory of Natural Medicines, Department of Chinese Medicines Analysis, China Pharmaceutical University, Nanjing, China; ^2^Key Laboratory of Modern Preparation of TCM, Ministry of Education, Jiangxi University of Traditional Chinese Medicine, Nanchang, China; ^3^Cellular and Molecular Biology Center of China Pharmaceutical University, Nanjing, China; ^4^State Key Laboratory of Natural Medicines, Jiangsu Key Laboratory of TCM Evaluation and Translational Research, Department of Complex Prescription of TCM, China Pharmaceutical University, Nanjing, China

**Keywords:** TGF-β1, HIF-1α, succinate, NLRP3 inflammasome, synovial fibrosis, collagen-induced arthritis, clematichinenoside AR

## Abstract

Clematichinenoside AR (C-AR) is a triterpene saponin isolated from the root of *Clematis manshurica* Rupr., which is a herbal medicine used in traditional Chinese medicine for the treatment of arthritis. C-AR exerts anti-inflammatory and immunosuppressive properties, but little is known about its action in the suppression of fibroblast activation. Low oxygen tension and transforming growth factor-β (TGF-β1) induction in the synovium contribute to fibrosis in arthritis. This study was designed to investigate the effect of C-AR on synovial fibrosis from the aspects of hypoxic TGF-β1 and hypoxia-inducible transcription factor-1α (HIF-1α) induction. In the synovium of rheumatoid arthritis (RA) rats, hypoxic TGF-β1 induction increased succinate accumulation due to the reversal of succinate dehydrogenase (SDH) activation and induced NLRP3 inflammasome activation in a manner dependent on HIF-1α induction. In response to NLRP3 inflammasome activation, the released IL-1β further increased TGF-β1 induction, suggesting the forward cycle between inflammation and fibrosis in myofibroblast activation. In the synovium of RA rats, C-AR inhibited hypoxic TGF-β1 induction and suppressed succinate-associated NLRP3 inflammasome activation by inhibiting SDH activity, and thereby prevented myofibroblast activation by blocking the cross-talk between inflammation and fibrosis. Taken together, these results showed that succinate worked as a metabolic signaling, linking inflammation with fibrosis through NLRP3 inflammasome activation. These findings suggested that synovial succinate accumulation and HIF-1α induction might be therapeutical targets for the prevention of fibrosis in arthritis.

## Introduction

Rheumatoid arthritis (RA) is an inflammatory autoimmune disease characterized by hyperplasia of the synovial membranes and progressive destruction of cartilage and bone with impaired joint function. In RA, fibroblast activation and extracellular matrix (ECM) remodeling are key events responsible for synovial tissue fibrosis (1, 2). Accumulating evidence has demonstrated the critical role of transforming growth factor-β (TGF-β) in fibrotic response. TGF-β is a secreted protein with multiple functions involved in the regulation of cell differentiation, tissue proliferation, and fibrogenesis. In response to inflammatory response, TGF-β1 drives myofibroblast activation through both canonical (Smad-based) and non-canonical (non-Smad-based) signaling pathways, leading to excessive ECM deposition and resultant fibrosis in synovium (3). Large amounts of TGF-β1 are stored in ECM accumulation; the activation and proliferation of myofibroblast correlate with a high expression of α-smooth muscle actin (α-SMA), which is considered myofibroblast marker (4). Similar to the action in diabetic nephropathy (5), pulmonary fibrosis (6), and liver fibrosis (7), TGF-β1 levels are elevated in the synovial fluid of arthritis patients, correlating with up-regulated genes involved in ECM turnover (8). Meanwhile, aberrant activation of TGF-β1 is observed in RA rats, and responsible for joint destruction (9). These events indicate that TGF-β1 is the control mediator in fibrogenesis.

Inflammation, oxidative stress, and alternation of immune parameters are often observed in the onset and persistence of RA (10, 11); however, hypoxia in the inflamed joint due to low oxygen tension emerges as an initial cause for these consequences in synovial fibrosis (12). In response to hypoxia, hypoxia-inducible transcription factor-1α (HIF-1α) serves as a hypoxia sensor to regulate cellular responses to adapt the anoxia environment. Meanwhile, as a transcription factor, HIF-1α induces an alternative transcriptional program, mainly enhancing gene expression encoding pro-inflammatory cytokines, growth factors, and ECM remodeling, to activate fibroblasts in special tissues, including adipose, liver, and kidney tissue (13–15). Inflammation induces fibroblast activation and fibrosis, while activation fibroblasts secrete pro-inflammatory cytokines and tissue-degrading enzymes to enhance inflammation and exacerbate tissue injury, forming a circle of regulation between inflammation and fibroblast activation. Although hypoxia and HIF-1α induction are observed in the synovium of arthritis (12, 16), the special role of HIF-1α with molecular signaling in fibrosis is still unknown.

Succinate, as an intermediate in citric acid cycle (CAC), is a universal metabolic signature response to hypoxia conditions (17). More recently, Tannahill et al. show that succinate induces IL-1β secretion through HIF-1α induction in macrophages (18). This finding elucidates that succinate might act as a metabolic signature to trigger inflammation under hypoxic conditions. As IL-1β maturation is mediated through NLRP3 inflammasome (19), hypoxic IL-1β production should be a result from NLRP3 inflammasome activation. In view of the important role of NLRP3 inflammasome in RA (20), we hypothesized that IL-1β might act as an inflammatory signaling for TGF-β1 activation in synovial fibrosis.

Clematichinenoside AR (C-AR) is a triterpene saponin isolated from the root of *Clematis manshurica* Rupr. As a herbal medicine, the root of *C. manshurica* Rupr. (Wei Ling Xian) has been used for the treatment of arthritis in traditional Chinese medicine with a prevalent clinical practice in many formulae defined in the Chinese Pharmacopoeia (2015 edition) and C-AR is considered the effective component for the treatment of RA (21). Previous study showed that C-AR exerts anti-inflammatory and immunosuppressive properties and ameliorate collagen-induced arthritis (CIA) in rats (22, 23). But up to now, little is known about its molecular targets and working pathways in the prevention of fibrosis. For these, we investigated the effects of C-AR on synovial fibrosis in CIA in rats with focus on the regulation of NLRP3 inflammasome activation and TGF-β1 induction from the aspects of succinate accumulation and HIF-1α induction. Our work demonstrated that succinate increased in synovial tissue due to low oxygen tension and acted as a metabolic signaling to induce TGF-β1 induction through NLRP3 inflammasome activation with the involvement of HIF-1α. C-AR inhibited succinate-mediated NLRP3 inflammasome activation by attenuating synovial hypoxia and HIF-1α induction, and thereby prevented fibroblast activation in CIA. These results suggested that synovial succinate accumulation and HIF-1α induction might be therapeutical targets for the prevention of fibrosis in RA.

## Materials and Methods

### Materials

Clematichinenoside AR was isolated from the roots of *C. manshurica* Rupr. as described previously (24), and the purity (>98%) was determined by high performance liquid chromatography (HPLC) analysis. The structure of C-AR was showed in Figure S1 in Data Sheet in Supplementary Material.

Complete Freund’s adjuvant (CFA) (#7001), incomplete Freund’s adjuvant (IFA) (#7002), and bovine type II collagen (#20021) were obtained from Chondrex, Inc. (Redmond, WA, USA). Recombinant Human TGF-β1 (PeproTech Inc., USA) and murine IL-1β (PeproTech Inc., USA) were dissolved in citric acid as a stock solution and then further diluted with PBS containing 5% trehalose before use. Digoxin and PX-478 were obtained from NRCCRM (Beijing, China) and Taizhou Rico Biological Technology Co., Ltd. (Taizhou, China), respectively. These agents were dissolved in dimethyl sulfoxide (DMSO) as a stock solution and the final working concentration of DMSO was <0.1% (v/v). Dimethyl malonate (V900555), dimethyl succinate (V900547), and aminooxyacetate (AOA, C13408) were provided by Sigma (St. Louis, MO, USA). The following items were purchased from the cited commercial sources: anti-IL-1β antibody (MAB201) (R&D, USA); anti-NLRP3 (NBP2-12446) and Novus Biologicals (Littleton, CO, USA); anti-α-SMA (ab32575), anti-cleaved caspase-1 (ab1872), anti-HIF-1α (ab51608), anti-TGF-β1 (ab64715), Goat Anti-Rabbit IgG H&L (Alexa Fluor 488) (ab181448), and Abcam (Cambridge, MA, USA); anti-GAPDH (AP0063), Goat Anti-Rabbit IgG (H+L) HRP (BS13278), and Bioworld Technology (St. Paul, MN, USA).

### Induction and Assessment of CIA and Drug Administration

Female Wistar rats (130–140 g, *n* = 50) were supplied by the Laboratory Animal Center of Yangzhou University (Yangzhou, China). Rats were housed under standard laboratory condition of constant temperature (23 ± 1°C) and 55 ± 5% relative humidity with a 12-h alternate light/dark cycle, fed with standard diet and tap water. Animal welfare and all experimental protocols were approved by the Animal Ethics Committee of China Pharmaceutical University.

To prepare CIA in the rat, on day 0, bovine type II collagen was emulsified in CFA (1:1, v/v) and then administrated intradermally into the base of rat tail, as the primary immunization. On day 7, rats were given a booster injection of bovine type II collagen emulsified in IFA (1:1, v/v), as the secondary immunization. After that, rats were administrated with C-AR (50 mg/kg, by gavage) or digoxin (100 μg/kg, by intraperitoneal injection) once a day for 2 weeks. The choice of dosage value for C-AR was based on the published reports (22, 23). Normal and model rats were orally given an equal volume of distilled water in parallel.

The clinical symptoms were recorded every 3 days since the secondary immunization, with arthritis index scores according to the following criteria: score 0, no swelling or inflammation; score 1, slight swelling and/or redness of paw; score 2, swelling in two or more joints; score 3, pronounced swelling with more than two joints; and score 4, severe swelling with joint rigidity. Meanwhile, paw swelling and body weight were recorded during the course, of which paw swelling was expressed as the volume of increase with respect to day 0 volume.

### Preparation of Rat Synovial Fibroblast and Cell Culture

Rat synovial fibroblasts were obtained as previously described (25). In brief, the isolated synovium was minced and digested with 4 mg/ml collagenase II (Sigma, USA) for 2 h at 37°C and then cultured in RPMI Medium 1640 until the cells outgrowing from the explants. Passages 3–6 of the synovial fibroblasts were used for the experiments.

To specifically suppress HIF-1α expression, NIH-3T3 cells (a cell line of embryo fibroblasts) were grown to 60% confluence and then transfected with small interfering RNA (siRNA) duplexes specific for HIF-1α (sc-35562, Santa Cruz Biotechnology) or control siRNA (sc-37007) by siRNA transfection reagent (sc-29528). After transfection, cells were cultured in Dulbecco’s Minimum Essential Medium (DMEM) for 24–48 h before experiments.

### Quantitative Real-time PCR Analysis

Total RNA from rat synovium was extracted with TRIzol Reagent (Beyotime Biotechnology Corporation, China), and cDNA was synthesized by an All-In-One-RT MasterMix synthesis kit (ABM, Canada), following the manufacturer’s instructions. cDNA was amplified by quantitative real-time PCR with the SYBR Green I PCR kit (BIO-RAD, USA) and the CFX96™ Realtime System (BIO-RAD, USA). Primer sequences are listed in Table S1 in Data Sheet in Supplementary Material. The mRNA level of individual genes was normalized to β-actin and calculated by 2^−ΔΔCt^ data analysis method (26).

### Biochemical Assay

Synovial tissue of RA was lysed in ice-cold RIPA buffer with Tissue Lyser, and then centrifuged at 13,000 *g* for 15 min at 4°C. The supernatant was collected. The contents of lactate and succinate and the activity of succinate dehydrogenase (SDH) in the supernatant were assayed with the commercial Kits, while IL-1β was measured with ELISA Kit (Neobioscience Technology Co., China). For the assay in cultured cells, confluent synovial fibroblasts or transfected NIH-3T3 cells were pretreated with indicated agents and then stimulated with TGF-β1 (10 ng/ml) or dimethyl succinate (5 mM) for 48 or 8 h. After incubation, lactate and IL-1β in the supernatant and succinate and SDH activity in the lysed cells were assayed as mentioned above.

### Western Blot Analysis

For protein analysis, synovial fibroblasts or transfected NIH-3T3 cells were lysed in ice-cold RIPA buffer and incubated in an ice bath for 45 min. Protein was obtained by centrifugation at 12,000 *g* for 20 min at 4°C and quantified with Bicinchoninic Acid Protein Assay Kit (Beyotime Biotechnology Corporation, China). Equal amount of protein was separated on polyacrylamide SDS gels and transferred to polyvinylidene difluoride (PVDF) membranes by electrophoresis, following incubation with the specific primary antibodies and HRP-conjugated secondary antibody. The values of band intensities were detected by enhanced chemiluminescence (ECL) and densitometry was analyzed quantitatively by Image-Pro Plus 6.0 (IPP 6.0) software.

### Histopathological and Immunohistochemical Examination

Synovial tissue were fixed with 4% (w/v) paraformaldehyde, embedded in paraffin, and then sectioned at 5-μm thickness. The sections were dewaxed using xylene and dehydrated in a gradient of alcohol. For the histological evaluation, the tissue slices were stained with hematoxylin and eosin (H&E) or Masson’s trichrome staining, according to standard protocols. For immunohistochemistry analysis, the sections were subjected to 3% hydrogen peroxide to quench the endogenous peroxidase activity, and then incubated with specific primary antibodies and HRP-conjugated secondary antibodies. Images were observed and captured with an optical microscope (Olympus, Japan).

### Pimonidazole Staining and Immunofluorescence

To investigate synovial hypoxia, rats were injected with hypoxyprobe (pimonidazole HCL) (Hypoxyprobe™-1 Plus Kit, Burlington, VT, USA) at a dosage of 60 mg/kg for 45 min prior to sacrifice. Immunoperoxidase staining for hypoxyprobe binding in a formalin-fixed paraffin embedded synovium section using 1:100 dilution of FITC-MAb1 and 1:100 dilution of horseradish peroxidase conjugated rabbit anti-FITC IgG. The image was observed by inverted fluorescence microscope (Nikon ECLIPSE Ti-s).

For cellular hypoxia, synovial fibroblasts were detected after adding hypoxyprobe (20 μM) and treated with C-AR or PX-478, then, exposed to hypoxia (1% O_2_, 4 h) or TGF-β (10 ng/ml, 48 h). For immunofluorescence assay, synovial fibroblasts were cultured on coverslips, pretreated with C-AR, and then stimulated with TGF-β1 (10 ng/ml, 48 h) or dimethyl succinate (5 mM, 8 h). After incubation, synovial fibroblasts were fixed with 4% paraformaldehyde in PBS for 15 min, and then blocked with 5% BSA containing 0.1% Triton X-100 for 60 min. Following incubation with primary antibodies and secondary antibodies, cells were visualized under a confocal scanning microscope (Zeiss LSM 700).

### Statistical Analysis

All of the experimental results are expressed as the mean ± SD (standard deviation), and each experiment was performed a minimum of four times. Data were analyzed by two-tailed *t*-test, or one-way ANOVA test with Student–Newman–Keuls test for comparison of two groups. A value of *p* < 0.05 was considered to be statistically significant.

## Results

### C-AR Alleviated TGF-β1 Expression and Synovial Fibrosis in CIA Rats

**Figure 1:**
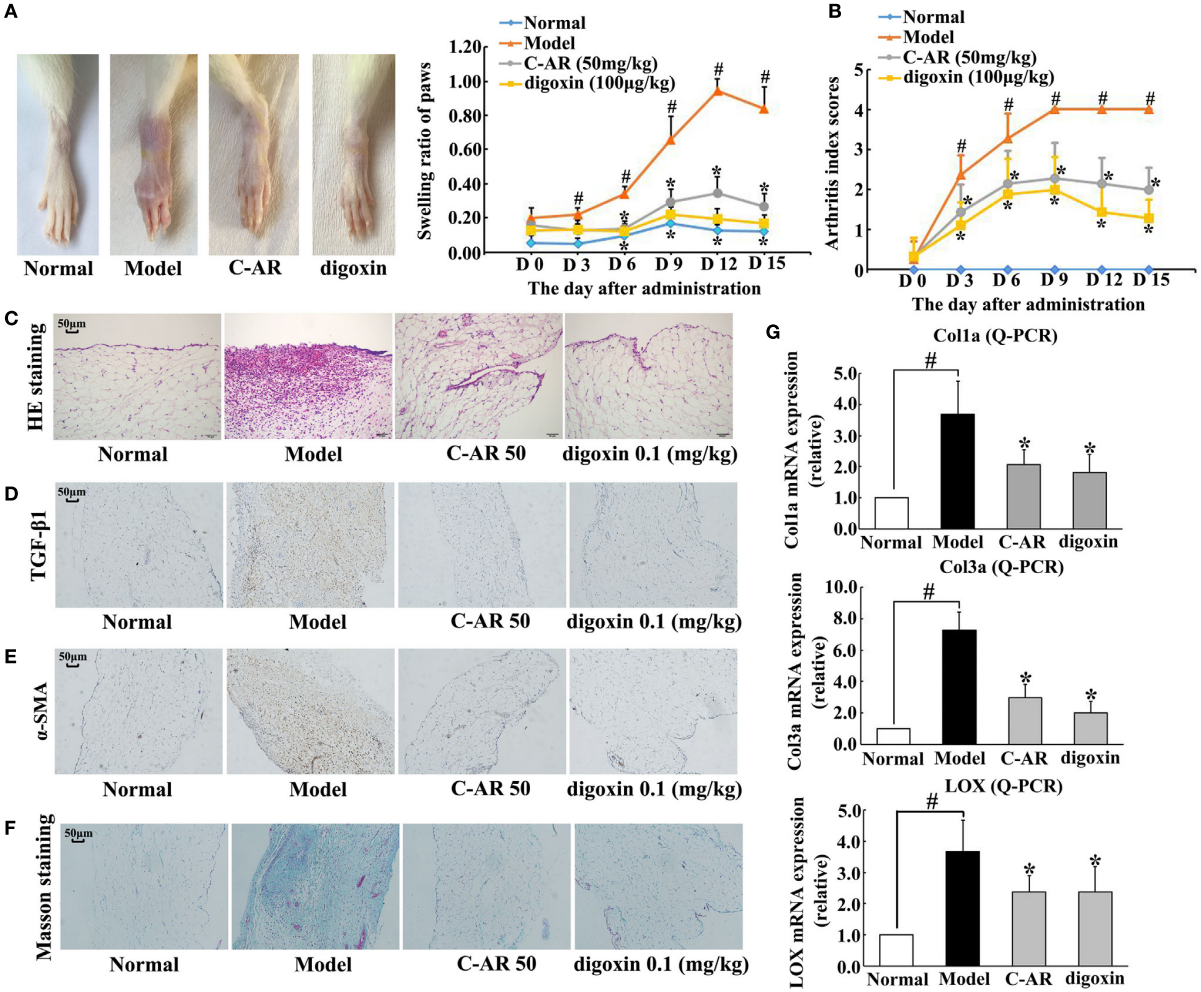
**C-AR attenuated synovial TGF-β1 expression and fibrosis in collagen-induced arthritis (CIA) rats**. **(A,B)** The severity of arthritis evaluated by the paw swelling **(A)** and the arthritis index scores **(B)** in rats. *p < 0.05 vs. the model; ^#^*p* < 0.05 vs. the normal (*n* = 8); **(C)** histopathological examination in the synovial tissue of CIA rats (original magnification, 100×; scale bar, 50 μm); **(D–F)** immunohistochemical staining of TGF-β1 and α-SMA and Masson’s trichrome staining in the synovial tissue of CIA rats (original magnification, 100×; scale bar, 50 μm); **(G)** the mRNA levels of Col1α, Col3α, and LOX in the synovial tissue of CIA rats (the data were expressed as the fold change relative to the normal). The results were expressed as the mean ± SD of four independent experiments. *p < 0.05 vs. the model; ^#^*p* < 0.05 vs. the indicated treatment.

Collagen challenge induced arthritis in rats, as evidenced by red swelling in the paw and functional impairment (Figures 1A,B). Administration of C-AR (50 mg/kg) and HIF-1α inhibitor digoxin (100 μg/kg) reduced swollen ratio and ameliorated the clinical symptoms indicated the reduced arthritis index scores (Figures 1A,B). Histological examination showed that C-AR and digoxin treatment ameliorated synovial hyperplasia and congestion with a reduction in the infiltration of inflammatory cells in the synovial tissue (Figure 1C). In addition, C-AR treatment improved body weight gain in the course of arthritis (Figure S2 in Data Sheet in Supplementary Material). These results well demonstrated the beneficial effects of C-AR on the amelioration of arthritis in rats.

Meanwhile, we examined fibrosis in the synovial tissues. Immunohistochemistry staining showed TGF-β1 and α-SMA overexpression in the synovial tissue, indicating fibroblast activation, whereas these alternations were prevented by C-AR and digoxin treatment (Figures 1D,E). Masson trichrome stain sections showed an attenuated fibrosis in C-AR or digoxin-treated CIA rats (Figure 1F). Coincidentally, C-AR treatment normalized gene expressions for Col1α, Col3α, and LOX in synovial tissue (Figure 1G). These results showed that C-AR prevented fibrosis in synovial tissue. Digoxin, a HIF-1α inhibitor, effectively reduced protein or gene expressions of TGF-β1, α-SMA, and ECM, indicative of the potential involvement of HIF-1α.

### C-AR Attenuated Hypoxia and Inhibited HIF-1α Induction in the Synovial Tissue of CIA Rats

**Figure 2:**
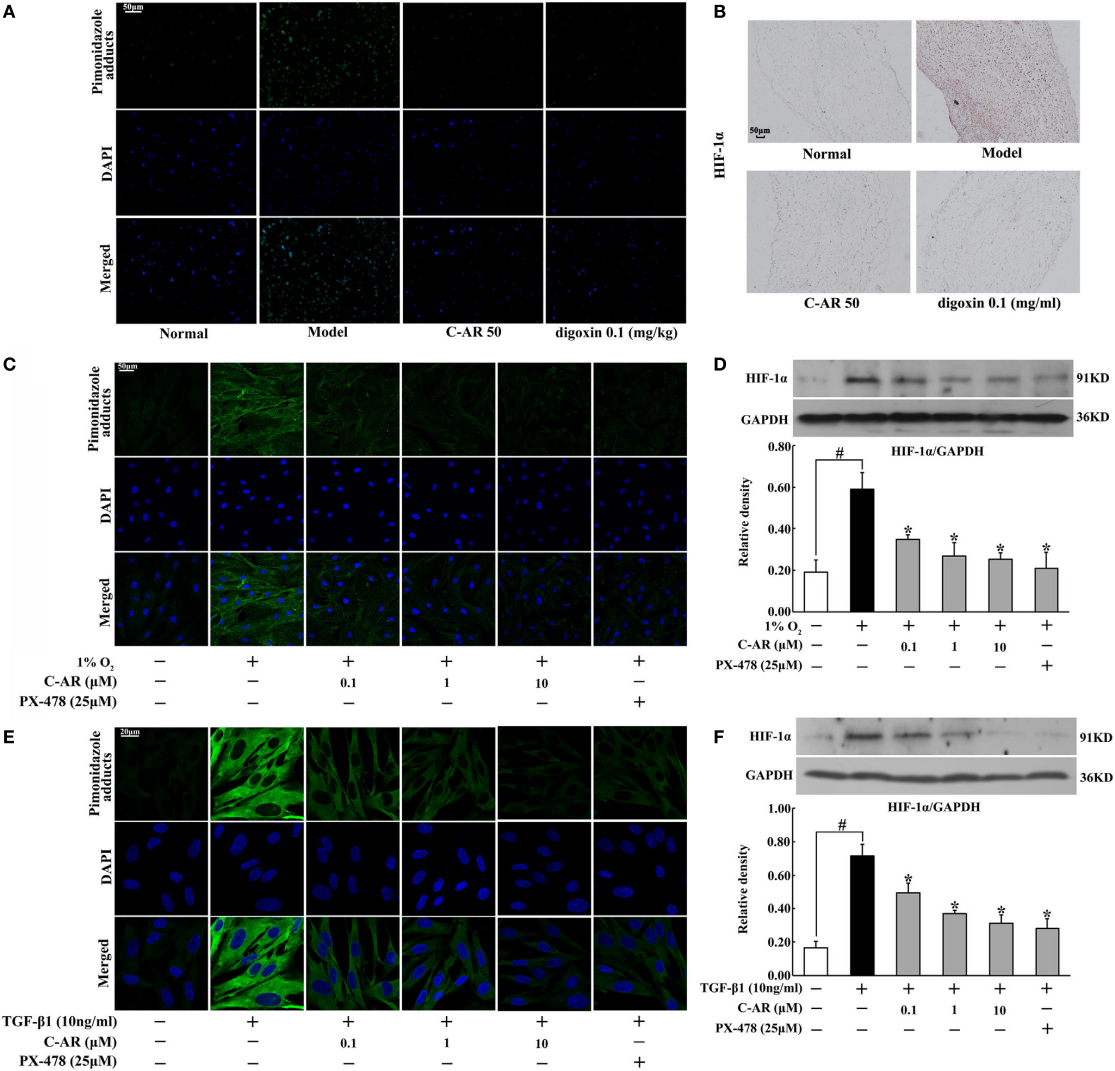
**C-AR alleviated hypoxia and suppressed HIF-1α expression in the synovial tissue of collagen-induced arthritis (CIA) rats**. **(A)** Pimonidazole staining in the synovial tissue of CIA rats (original magnification, 200×; scale bar, 50 μm); **(B)** immunohistochemical staining of HIF-1α in the synovial tissue of CIA rats (original magnification, 100×; scale bar, 50 μm); **(C)** synovial fibroblasts were cultured under hypoxic conditions (1% O_2_) for 24 h and pimonidazole staining was viewed under a confocal scanning microscope (original magnification, 630×; scale bar, 50 μm); **(D)** HIF-1α expression in synovial fibroblasts cultured under hypoxic conditions (1% O_2_) for 24 h; **(E,F)** pimonidazole staining **(E)** and HIF-1α expression **(F)** in synovial fibroblasts exposed to TGF-β1 (original magnification, 630×; scale bar, 20 μm). Data were expressed as the mean ± SD from four independent experiments. *p < 0.05 vs. the model; ^#^*p* < 0.05 vs. the indicated treatment.

Accompanied with fibrosis, hypoxia also occurred in synovial tissue, indicated by increased staining of hypoxia adducts with pimonidazole (Figure 2A). C-AR and digoxin treatment attenuated hypoxia and then inhibited HIF-1α protein accumulation in the synovium of CIA rats (Figures 2A,B). Meanwhile, HIF-1α gene expression was also reduced in the synovium (Figure S3 in Data Sheet in Supplementary Material). Similarly, C-AR treatment effectively attenuated hypoxia and reduced HIF-1α accumulation at concentrations ranging from 0.1 to 10 μM in synovial fibroblasts exposed to 1% O_2_ (Figures 2C,D). HIF-1α inhibitor PX-478 showed a similar regulatory tendency as C-AR. TGF-β1 stimulation increased HIF-1α induction with increased pimonidazole staining in synovial fibroblasts, indicating that TGF-β1-induced HIF-1α induction in a manner of low oxygen tension-dependent (Figures 2E,F). C-AR as well as PX-478 prevented TGF-β1-induced low oxygen tension in fibroblasts, and thereby inhibited HIF-1α induction with reduced lactate accumulation (Figure S3 in Data Sheet in Supplementary Material). Meanwhile, C-AR treatment reduced lactate accumulation in the synovial tissue and fibroblasts (Figure S4 in Data Sheet in Supplementary Material). These results *in vivo* and *in vitro* demonstrated that C-AR reduced HIF-1α accumulation by preventing low oxygen tension.

### C-AR Reduced Succinate Accumulation in Synovial Tissue

**Figure 3:**
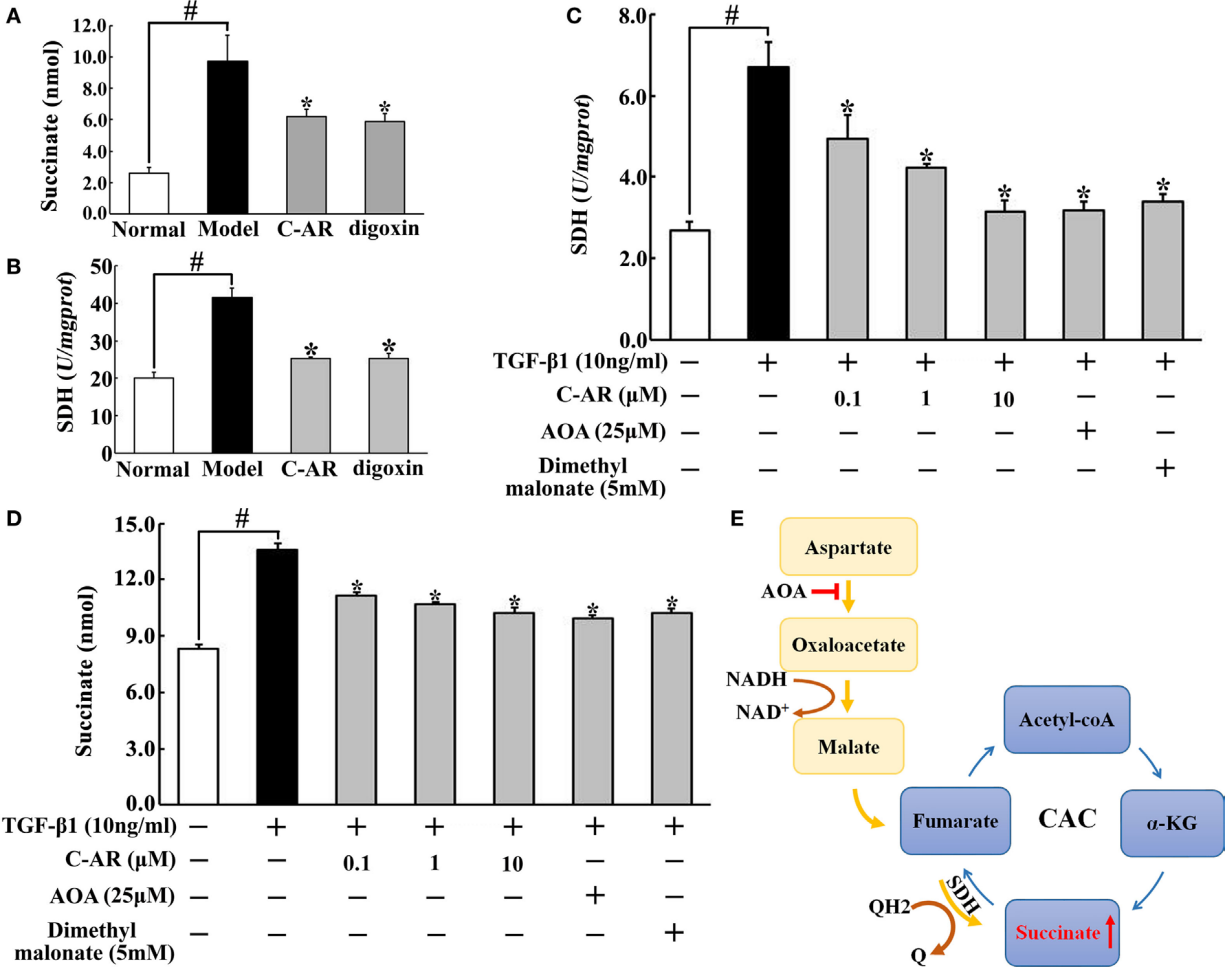
**C-AR reduced succinate accumulation in synovial tissue**. **(A,B)** Succinate abundance **(A)** and succinate dehydrogenase (SDH) activity **(B)** in synovial tissue of collagen-induced arthritis (CIA) rats; **(C,D)** SDH activity **(C)** and the level of succinate **(D)** in synovial fibroblasts stimulated with TGF-β1; **(E)** proposed pathway for reversal succinate accumulation. The results all above were expressed as the mean ± SD (*n* = 4). *p < 0.05 vs. the model; ^#^*p* < 0.05 vs. the indicated treatment.

Accompanied with local hypoxia, succinate accumulated in the synovium of CIA rats (Figure 3A). C-AR and digoxin treatment reduced succinate accumulation with inhibition of SDH activity (Figures 3A,B). Consistent with the regulation *in vivo*, C-AR treatment inhibited SDH activity and reduced succinate accumulation in a concentration-dependent manner in synovial fibroblasts when exposed to TGF-β1 treatment (Figures 3C,D). In CAC, SDH converts succinate to fumarate, inhibition of SDH should increase succinate accumulation. However, we found that SDH inhibitor dimethyl malonate inhibited TGF-β1-induced succinate production in fibroblasts. In view of the increased SDH activity, this result suggested that SDH might act in reverse for succinate formation. As an inhibitor of malate/aspartate shuttle (MAS), AOA reduced succinate accumulation with inhibited SDH activity, suggesting that MAS pathway might provide substrate for the reversal succinate production (Figure 3E).

### C-AR Inhibited NLRP3 Inflammasome Activation in Synovial Tissue

**Figure 4:**
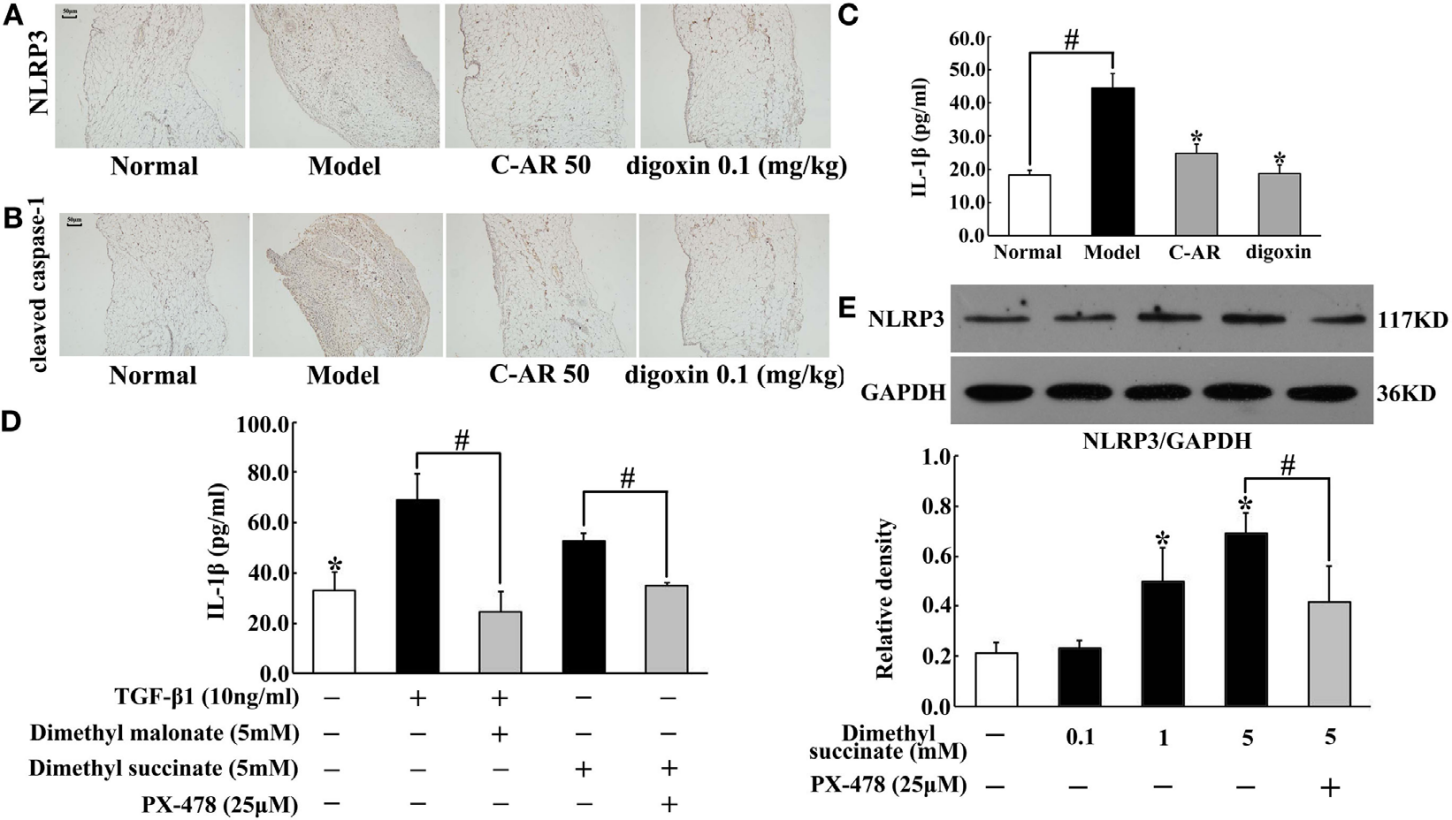
**C-AR inhibited NLRP3 inflammasome activation in synovial tissue**. **(A,B)** Immunohistochemistry staining of NLRP3 expression **(A)** and cleaved caspase-1 activation **(B)** in the synovial tissue of collagen-induced arthritis (CIA) rats (original magnification, 100 × ; scale bar, 50 μm). **(C,D)** IL-1β in the synovial tissue of CIA rats **(C)** and TGF-β1-stimulated synovial fibroblasts **(D)** were measured by ELISA Kits (*n* = 6); **(E)** NLRP3 protein expression in succinate-stimulated synovial fibroblasts. The results were derived from four independent experiments for immunohistochemistry staining and Western blot and expressed as the mean ± SD. *p < 0.05 vs. the model; ^#^*p* < 0.05 vs. the indicated treatment.

We observed increased IL-1β production accompanied with NLRP3 and cleaved caspase-1 overexpression in synovial tissue (Figures 4A–C). C-AR and digoxin administration attenuated NLRP3 and cleaved caspase-1 expression, and then reduced IL-1β production (Figures 4A–C), which was indicative of the role in suppressing NLRP3 inflammasome activation. As succinate is documented to promote IL-1β in macrophages, we investigated the possible implication of succinate in synovial IL-1β production. As shown in Figure 4D, SDH inhibitor dimethyl malonate inhibited TGF-β1-induced IL-1β production, indicating the involvement of succinate. Dimethyl succinate is a cell permeable derivative of succinate readily taken up by cells, where it is then amplified thereby increasing succinate level. Similar to TGF-β1, succinate also increased IL-1β production, despite less extent than TGF-β1 (Figure 4D). In line with the action, we observed succinate enhanced NLRP3 induction in a concentration-dependent way (Figure 4E). These results suggested that TGF-β1-induced NLRP3 inflammasome activation through succinate, at least in part. HIF-1α inhibitor PX-478 diminished the enhanced effect of succinate on IL-1β production and NLRP3 expression, suggesting the potential involvement of HIF-1α in NLRP3 inflammasome activation (Figures 4D,E).

### Succinate Induced NLRP3 Inflammasome Activation *via* HIF-1α

**Figure 5:**
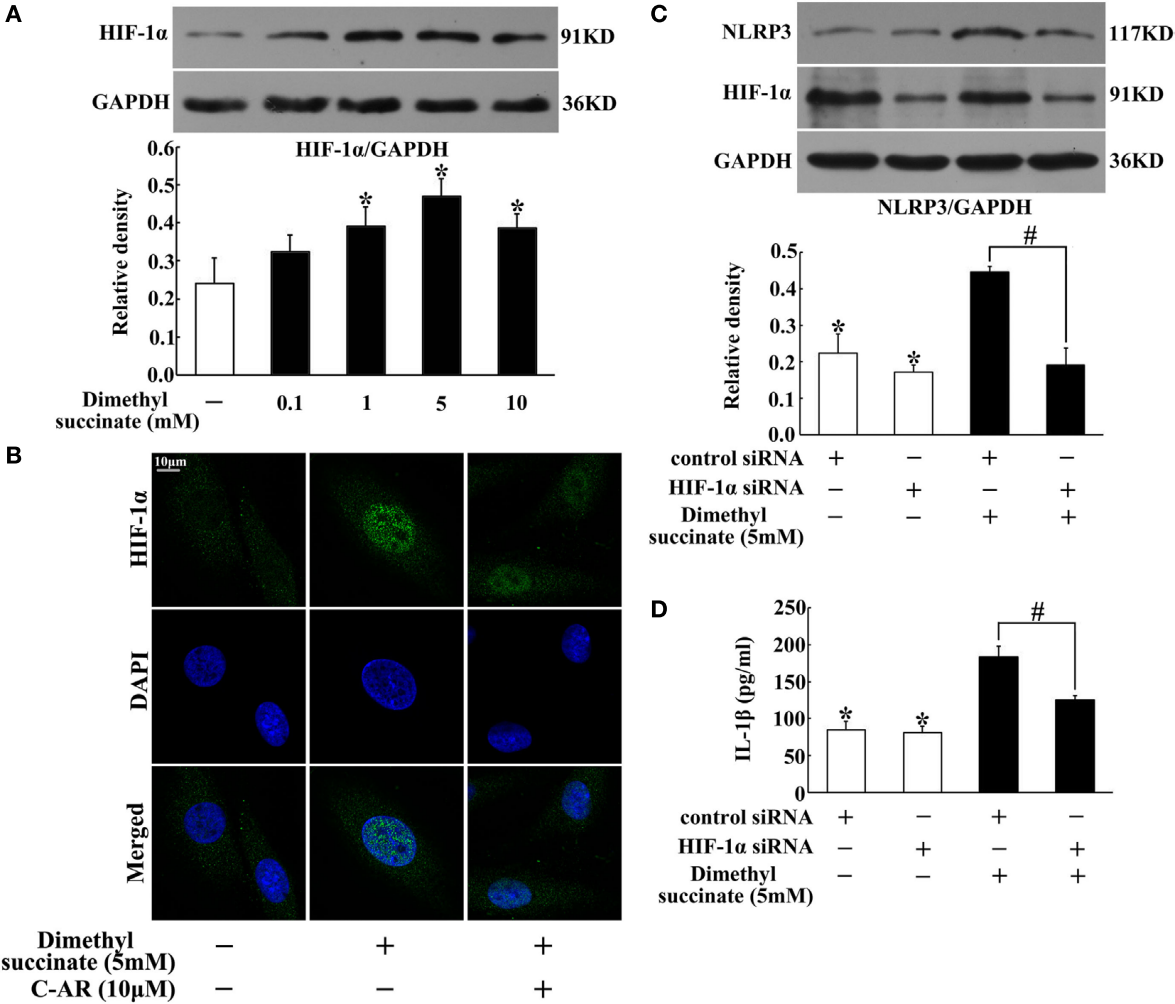
**Succinate induced NLRP3 inflammasome activation *via* HIF-1α**. **(A)** HIF-1α expression in succinate-stimulated synovial fibroblasts. *p < 0.05 vs. the normal control group. **(B)** HIF-1α translocation in succinate-stimulated synovial fibroblasts was viewed under a confocal scanning microscope (original magnification, 630×; scale bar, 10 μm); **(C,D)** NLRP3 expression **(C)** and IL-1β **(D)** production in transfected NIH-3T3 cells (HIF-1α siRNA). The results above were derived from four independent experiments and expressed as the mean ± SD for ELISA (*n* = 6). *p < 0.05 vs. the HIF-1α siRNA; ^#^*p* < 0.05 vs. the indicated treatment.

In synovial fibroblasts, succinate increased HIF-1α production (Figure 5A). HIF-1α was distributed in the cytosol, and in response to succinate stimulation, HIF-1α accumulation was accompanied by its translocation to the nucleus (Figure 5B). To directly address the functional interaction between HIF-1α and NLRP3 inflammasome activation, we investigated NLRP3 expression and IL-1β secretion in NIH-3T3 cells, a cell line of an embryo fibroblast, by transfecting them with siRNA of HIF-1α. Knockdown of HIF-1α diminished the enhancing effects of succinate on NLRP3 expression and IL-1β secretion (Figures 5C,D), indicating that HIF-1α induction was required for succinate-associated NLRP3 inflammasome activation.

### C-AR Attenuated Synovial Fibroblast Activation with Regulation IL-1β Secretion

**Figure 6:**
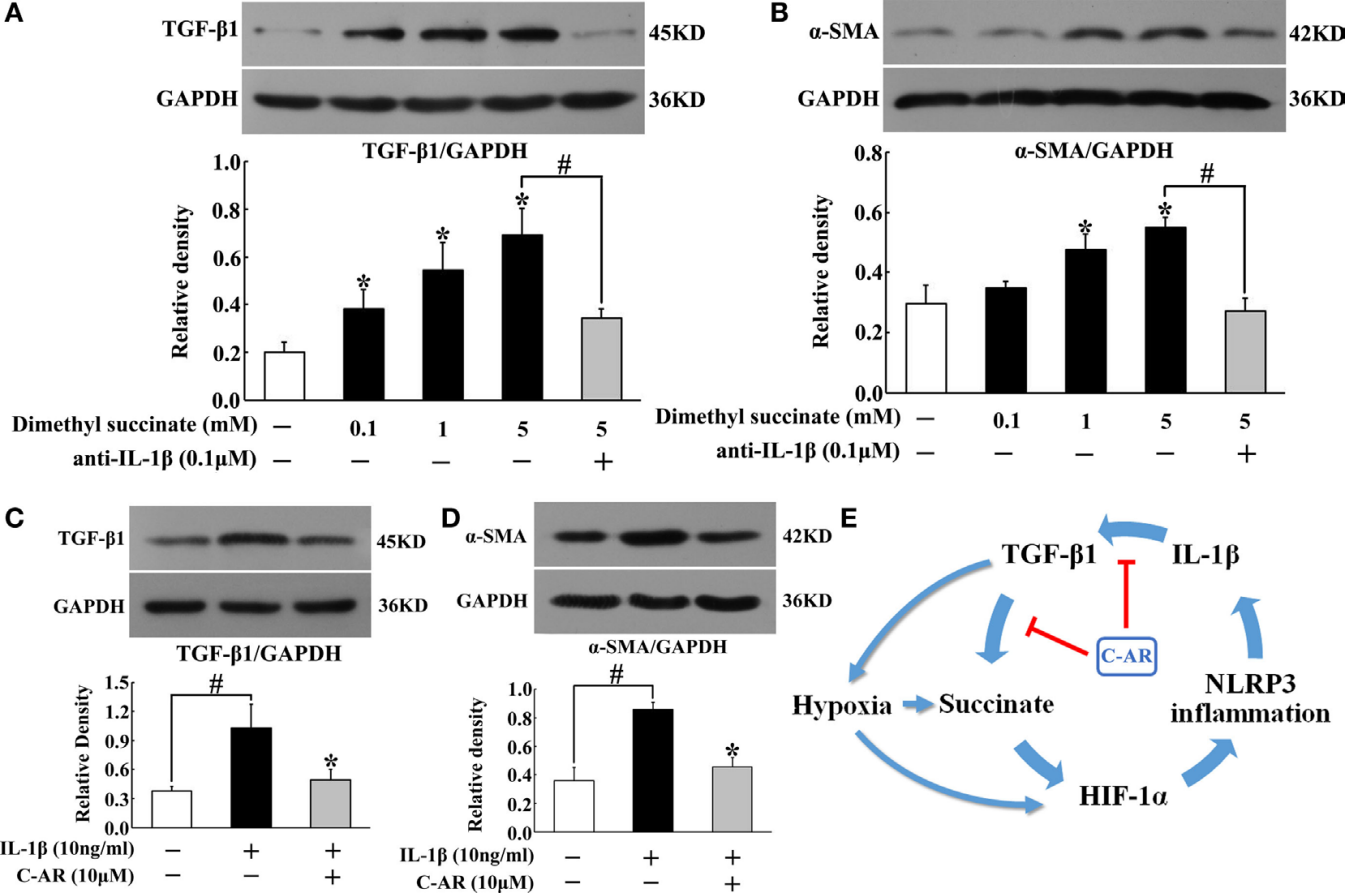
**TGF-β1 and α-SMA induction in synovial fibroblasts**. **(A,B)** TGF-β1 and α-SMA expression in succinate-stimulated synovial fibroblasts; **(C,D)** TGF-β1 and α-SMA in IL-1β-stimulated synovial fibroblasts. The results all above are expressed as the mean ± SD of four independent experiments. *p < 0.05 vs. the model group; ^#^*p* < 0.05 vs. the indicated treatment. **(E)** The proposed pathway for C-AR action in the suppression of synovial fibrosis.

To know the contribution of succinate and NLRP3 inflammasome activation to synovial fibroblasts activation, we observed TGF-β1 and α-SMA protein expression in fibroblasts exposed to dimethyl succinate. Succinate challenge increased TGF-β1 and α-SMA expression, whereas these increased expressions were reduced by neutralizing IL-1β with anti-IL-1β antibody treatment (Figures 6A,B), suggesting the potential role of IL-1β in succinate-mediated fibroblasts activation. For further confirmation, we observed the expression of TGF-β1 and α-SMA in fibroblasts to IL-1β stimulation. As shown in Figures 6C,D, incubation of fibroblasts with IL-1β resulted in TGF-β1 and α-SMA proteins accumulation, and these alternations were reversed by pretreatment with C-AR. Together with above-mentioned results, they indicated that succinate worked as a metabolic signaling, amplifying TGF-β1 action in fibroblasts through NLRP3 inflammasome activation. C-AR reduced succinate accumulation and thereby blocked the forward cycle toward sustained fibroblast activation (Figure 6E).

## Discussion

Inflammation and fibrosis persistently exist in the pathological progress of RA. In the present study, we demonstrated that IL-1β-mediated inflammation led to fibroblast activation through TGF-β1 induction, which further stimulated IL-1β production for the persistent fibroblast activation. Succinate accumulation acted as a metabolic signaling linking IL-1β and TGF-β1 induction in the ahead cycle, establishing the cross-talk between inflammation and fibrosis and further exacerbating tissue injury in RA.

Rheumatoid arthritis is characterized by synovial proliferation and invasiveness, in which fibroblast activation links inflammation to ECM remodeling, contributing to fibrosis and joint injury. C-AR reduced joint swelling with functional improvement in arthritis rats, and the therapeutic effects should be related to the suppression of synovial invasiveness, as we observed that C-AR ameliorated synovial hyperplasia and congestion in RA rats. TGF-β1 is considered to be the master regulator of fibrosis, because it activates myofibroblasts, promotes expression of ECM genes, and inhibits ECM degradation (3). As a cellular contractile apparatus, α-SMA induction is a marker of myofibroblast formation. C-AR suppressed TGF-β1 and α-SMA induction, and thus prevented ECM deposits in the synovium. C-AR is documented to exert anti-inflammatory activity in RA rats (22, 23); the present work further showed that C-AR reduced ECM deposits *via* suppression of myofibroblast activation, elucidating the special role of C-AR in anti-fibrosis.

Hypoxia-inducible transcription factor-1α inhibitor digoxin inhibited myofibroblast activation in the synovium, indicating the potential role of HIF-1α in RA. Consistently, we observed low oxygen tension in the synovium. The dysregulated architecture of the microvasculature in synovial invasiveness and the increased demand of oxygen during the inflammatory response should be the cause of synovial hypoxia (27). C-AR as well as digoxin inhibited inflammation and prevented fibrosis, and these actions should attenuate hypoxia by reducing oxygen demand in the synovium. Interestingly, we found that accompanied with low oxygen tension, succinate accumulated in the synovium. Succinate is an intermediate of the mitochondrial CAC and recent studies demonstrated the special role of succinate in inflammation (18, 28). In CAC, SDH catalyzes succinate to produce fumarate, and theoretically, inhibition of SDH should lead to succinate accumulation. Indeed, SDH inhibition induces succinate accumulation in tumor cells (29). However, in the present study, we observed succinate accumulation accompanied with increased SDH activity. SDH inhibitor dimethyl malonate reduced succinate accumulation, indicating that succinate accumulation should be a result of the reversal of SDH activity. In ischemic heart, succinate accumulation is proposed to be a result of the reversed SDH activation due to the supply of fumarate from the MAS (30). Consistent with the hypothesis, MAS inhibitor AOA suppressed SDH activity and reduced succinate accumulation in TGF-β1-stimulated synovial myofibroblasts. C-AR inhibited SDH activity and thereby prevented succinate accumulation. In macrophages, LPS-induced succinate accumulation is proposed to be a consequence of glycolytic reprograming (18, 28); our work showed that in myofibroblasts, TGF-β1 increased succinate accumulation through the reversal of SDH, which might be the alternative way for inflammatory succinate production.

In RA rats, we observed TGF-β1 induction in the synovium and showed that TGF-β1 promoted succinate accumulation in myofibroblast. In macrophages, succinate promotes IL-1β production in a manner dependent on HIF-1α (18). As IL-1β is a crucial mediator of the inflammatory response and plays an important role in arthritis (31), it is tempted to know if succinate contributed to IL-1β production in activated fibroblasts. In the synovium of RA rats, NLRP3 induction and increased IL-1β production suggested the potential implication of succinate. NLRP3 inflammasome complex is assembled by NLRP3, the adaptor protein ASC and caspase-1, and the action is to promote IL-1β maturation and secretion through cleavage by caspase-1 (20). In myofibroblasts, succinate induced NLRP3 expression with an increase of IL-1β production, indicating inflammasome activation. In tumor cells, succinate is demonstrated to stabilize HIF-1α protein by inhibiting proteasomal degradation (30). Consistently, in the present study succinate increased HIF-1α accumulation and knockdown of HIF-1α diminished its enhancing effects on NLRP3 induction and IL-1β production, indicating that succinate induced IL-1β production through NLRP3 inflammasome activation in a manner dependent of HIF-1α. Recently, Vande Walle et al. demonstrated NLRP3 inflammasome activation in RA (22), and our work further elucidated the special role of succinate and HIF-1α in NLRP3 inflammasome activation. C-AR reduced succinate accumulation and inhibited HIF-1α induction, thereby preventing NLRP3 inflammasome activation in the synovium of RA rats. Previous works demonstrated the anti-inflammatory effects of C-AR on RA rats (22, 23), and our work indicated that suppression of inflammasome activation should be the unifying mechanism for the same action.

Inflammatory response and fibrosis occur simultaneously in the invasive synovial tissue of RA and it is generally considered that TGF-β1 is a crucial mediator linking inflammation to fibroblast activation (3, 9). In response to inflammation, TGF-β1 induces myofibroblast activation, leading to fibrosis. RA is a progressive inflammatory disease, and here we showed that succinate/HIF-1α signaling should be the important cause for persistent TGF-β1 activation. In addition to reprogram adaptive response for anoxia metabolism, HIF-1α exerts the ability to induce fibrosis in special tissues (13–15). Here, we demonstrated the involvement of NLRP3 inflammasome activation, because HIF-1α was essential for succinate-mediated NLRP3 inflammasome activation, which further promoted TGF-β1 and α-SMA induction through IL-1β action. In myofibroblasts, TGF-β1 increased hypoxic succinate accumulation and subsequent NLRP3 inflammasome activation, indicating that TGF-β1 was an initial cause for IL-1β production, responsible for inflammatory response. As an important inflammatory mediator, IL-1β in turn further enhanced TGF-β1 activation, demonstrating the positive feedback regulation of fibroblast activation. In view of the special role of TGF-β1 and IL-1β in RA, the reciprocal regulate established a cycle or cross-talk between inflammation and fibrosis. In the forward cycle, succinate worked as a metabolic signaling responsible for sustained TGF-β1 induction, contributing to fibrosis in the synovial tissue of RA. C-AR blocked the cross-talk between IL-1β and TGF-β1 by reducing succinate accumulation, and thereby inhibited inflammation and prevented fibrosis in synovial tissue of RA.

Taken together, our work showed that anoxic succinate accumulation induced NLRP3 inflammasome activation through HIF-1α induction, then exacerbated inflammation and fibrosis in the synovium of RA rats. C-AR reduced succinate accumulation by attenuating synovial hypoxia and then inhibited inflammation and fibrosis by preventing NLRP3 inflammasome activation. Consistent with the published studies which show that succinate accumulated in the synovial fluids from RA patients (32, 33), the finding of the role of succinate/NLRP3 inflammation signaling not only provides insight into the connection between inflammation and fibroblast activation but also reveals therapeutic targets for RA treatment. Moreover, our work presented a potential therapeutic strategy for the application of C-AR in the management of RA.

## Author Contributions

Concept and design: Yi L, B-LL, G-ZX, and L-FL. Acquisition of data: Yi L, J-YZ, Yang L, and CW. Analysis and interpretation: Yi L, J-YZ, J-QL, JY, and X-NM. Drafting and editing of the manuscript: Yi L, B-LL, G-ZX, and L-FL. All the authors read and approved the final manuscript.

## Conflict of Interest Statement

The authors declare that the research was conducted in the absence of any commercial or financial relationships that could be construed as a potential conflict of interest.
