# Supplementary material for: Succinate/NLRP3 Inflammasome Induces Synovial Fibroblast Activation: Therapeutical Effects of Clematichinenoside AR on Arthritis
Source: Front Immunol. 2016 Dec 7;7:532. doi: 10.3389/fimmu.2016.00532 (PMC5141240; doi:10.3389/fimmu.2016.00532)
Supplement: Supplementary file 1 [file Data_Sheet_1.DOCX]

**Supplement Table 1.** The primer sequences of RT-PCR

| Gene | Primer sequence |
| --- | --- |
| Col1α  Col3α  LOX  HIF-1α  β-actin | forward primer, 5'-CCGGCTCCTGCTCCTCTTA-3'  reverse primer, 5'-AGGGACCCTTAGGCCATTGT-3'  forward primer, 5'-GCCTCCCAGAACATTACATACC-3'  reverse primer, 5'-AGACTGTCTTGCTCCATTCACC-3'  forward primer, 5'-TATGGCACCGGTTACTTCCAGTA-3'  reverse primer, 5'-ACGTGGATGCCTGGATGTAGT-3  forward primer, 5'-TCAAGTCAGCAACGTGGAAG-3  reverse primer, 5'-TATCGAGGCTGTGTCGACTG-3  forward primer, 5'-TACTGCCCTGGCTCCTAGC-3 |
|  | reverse primer, 5'-AGAGCCACCAATCCACACA-3 |

**

**

Molecular formula: C82H134O43

Molecular weight: 1806

**Supplement Figure 1** The structure of clematichinenoside AR (C-AR).


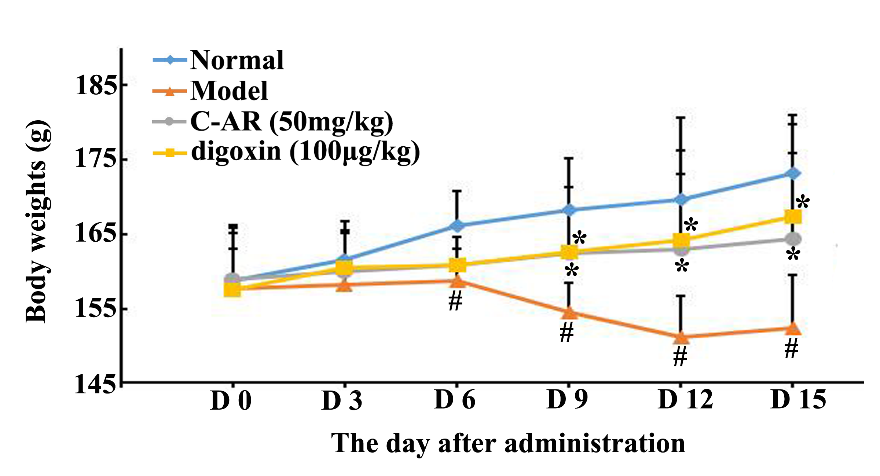


**Supplement Figure 2** Clematichinenoside AR (C-AR) improved body weight gain in collagen-induced arthritis rats. **p < 0.05* *vs*. the model; ^#^*p < 0.05 vs*. the normal (*n=8*).


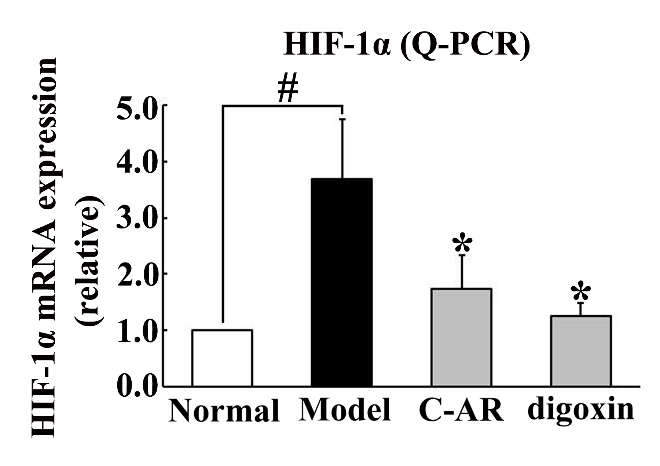


**Supplement Figure 3** Clematichinenoside AR (C-AR) inhibited HIF-1α gene expression in synovial tissue of collagen-induced arthritis rats. The results were expressed as the mean ± SD of four independent experiments.**p < 0.05* *vs*. the model; ^#^*p < 0.05 vs*. the indicated treatment.


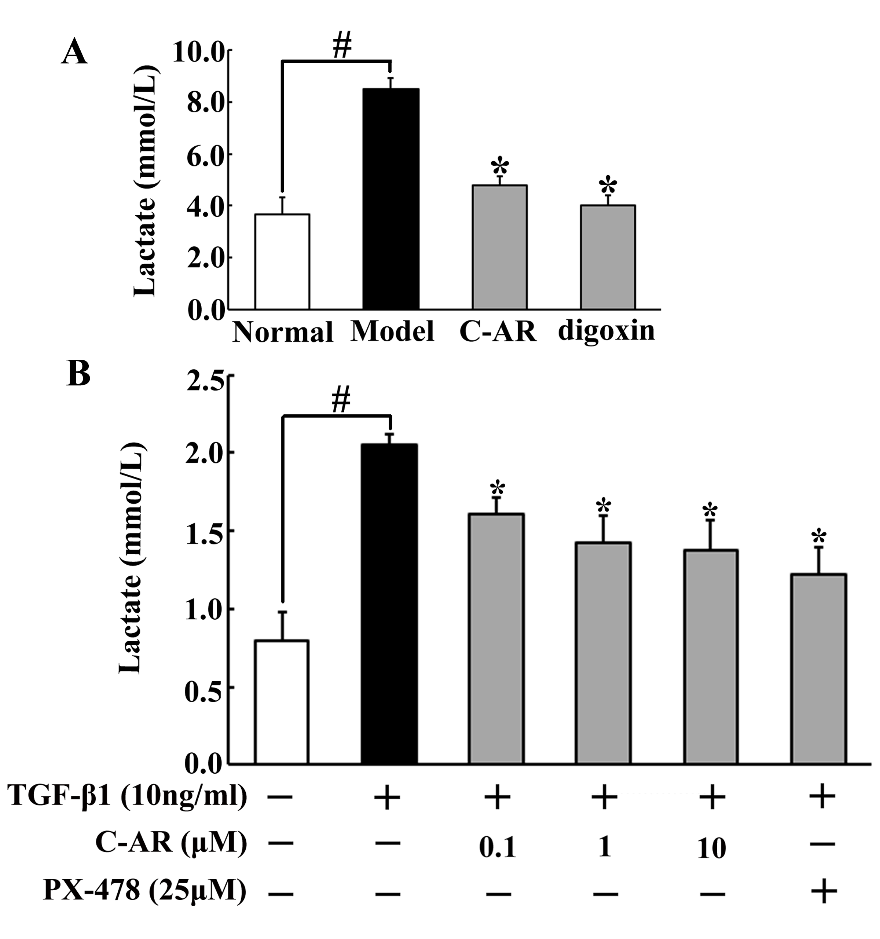


**Supplement Figure 4** Clematichinenoside AR (C-AR) reduced lactate accumulation. (**A**) Lactate in the synovial tissue of collagen-induced arthritis rats; (**B**) Lactate in TGF-β1-stimulated synovial fibroblasts. The results were expressed as the mean ± SD of four independent experiments.**p < 0.05* *vs*. the model; ^#^*p < 0.05 vs*. the indicated treatment.
